# Giant Negative T Waves and QT Prolongation in Non-cardiogenic Pulmonary Edema: A Case Report and Review of Literature

**DOI:** 10.7759/cureus.3423

**Published:** 2018-10-08

**Authors:** Abdallah M Mansour, Obai Abdullah, Haytham Allaham, Cristina Danila, Sudarshan Balla

**Affiliations:** 1 Department of Internal Medicine, University of Missouri – Columbia, Columbia, USA; 2 Department of Internal Medicine, University of Missouri-Columbia, Columbia, USA

**Keywords:** t wave inversion, prolonged qt, pulmonary edema, wellen’s ecg

## Abstract

Giant negative T-waves have been linked to several cardiac and non-cardiac conditions. However, the presence of giant negative T-waves with QT prolongation in the setting of non-cardiogenic pulmonary edema is a rarely reported, female predominant, and poorly understood electrocardiographic phenomenon.

We report a case of a 28-year-old white female who presented with acute diarrhea and was admitted due to acute kidney injury caused by a hemolytic uremic syndrome (HUS). She was managed with multiple blood product transfusions, plasma exchange, and hemodialysis. Subsequently, she developed acute pulmonary edema requiring intubation and urgent hemodialysis. During this acute event, a unique electrocardiographic finding of anterolateral giant negative T-wave and QT prolongation progressively developed and began resolving with the resolution of the pulmonary edema. In addition to our case, 12 cases were reported upon review of the literature with similar electrocardiography (ECG) findings in the setting of non-cardiogenic, non-ischemic pulmonary edema.

Giant negative T-waves can be associated with non-cardiac pulmonary edema. Recognition of this rare Wellen’s-like electrocardiographic pattern in a patient without cardiac ischemia is crucial, especially in young females. Basic science and clinicopathological correlation studies are needed to understand the pathophysiology and prognosis behind these ECG findings.

## Introduction

T wave inversion is a well-known electrocardiographic finding that can be a normal variant or linked to cardiac and non-cardiac conditions [1]. Even though ischemic causes of T-wave inversion are very well reported and studied [2], non-ischemic and non-cardiac causes exist and physicians need to be aware of these non-ischemic etiologies.

We present a case of 28-year-old female who was admitted with hemolytic uremic syndrome (HUS)-induced renal failure. This was complicated with pulmonary edema requiring mechanical ventilation, hemodialysis, and was associated with giant negative T wave and corrected QT (QTc) prolongation. The purpose of our study is to illustrate this finding of giant negative T wave inversion in a young, previously healthy female who developed non-cardiogenic pulmonary edema without underlying cardiovascular factors. We also want to add our case to the 12 previously reported cases to raise awareness about this characteristic electrocardiogram (ECG) change associated with non-cardiogenic pulmonary edema, especially in females.

## Case presentation

A 28-year-old white female without a significant past medical history presented with abdominal pain, nausea, vomiting, and bloody diarrhea without fever, chills, or altered mentation; there was also no dyspnea or chest pain. Her family history was unremarkable. Physical exam revealed pallor, diffuse petechial rash, and generalized abdominal tenderness with an unremarkable neurological exam. Cardiac auscultation revealed normal heart sounds with no murmurs or S3. Her temperature was 37.2 °C, heart rate 90 beats per minute, blood pressure 121/87 mmHg, and an oxygen saturation of 99% on room air. Lab values on admission are shown in Table 1. A peripheral smear showed schistocytes, and stool studies were remarkable for Shiga toxin-producing E. coli. A diagnosis of HUS secondary to Shiga toxin-producing E. coli was made and she was admitted to the Medical Intensive Care Unit. She underwent supportive care with plasma exchange and transfusion of blood products.

**Table 1:** Lab values at admission ANC: absolute neutrophil count; BUN: blood urea nitrogen; Cl-: chloride; HCO_3-_: bicarbonate; INR: international normalized ratio; K+: potassium; LDH: lactate dehydrogenase; MCV: mean corpuscular volume; Na+: sodium; PTT: partial thromboplastin time; WBC: white blood cells

Lab test (unit)	Result	Reference value
Complete blood count		
Hemoglobin (g/dL)	7.7	12 - 15.5
Hematocrit (%)	25.6	34.9 - 44.5
MCV (fL)	81.5	81.6 - 98.3
WBC (× 10^9^/L)	6.65	3.5 - 10.5
ANC (× 10^9^/L)	4.87	1.7 - 7
Platelets count (x10^9^/L)	17	150 - 450
Reticulocytes (%)	6.7	0.5 - 1.81
Complete metabolic panel and miscellaneous		
Creatinine (mg/dL)	5.3	0.5 - 1.2
BUN (mg/dL)	63	6.0 - 20
Na+ (mmol/L)	139	136 - 145
K+ (mmol/L)	4.3	3.5 - 5.1
Cl- (mmol/L)	97	92 - 107
HCO_3-_ (mmol/L)	28	22 - 29
Ionized calcium (mmol/L)	1.21	1.12 - 1.3
Ionized magnesium (mmol/L)	0.52	0.43 - 0.61
Lactate (mmol/L)	0.7	0.5 - 2.2
LDH (units/L)	1650	135 - 214
Total bilirubin (mg/dL)	2.37	0.0 - 1.6
Indirect bilirubin (mg/dL)	1.8	0.1 - 1.2
INR	1.1	0.9 - 1.1
PTT (seconds)	28.7	25.7 - 35.2

**Figure 1:**
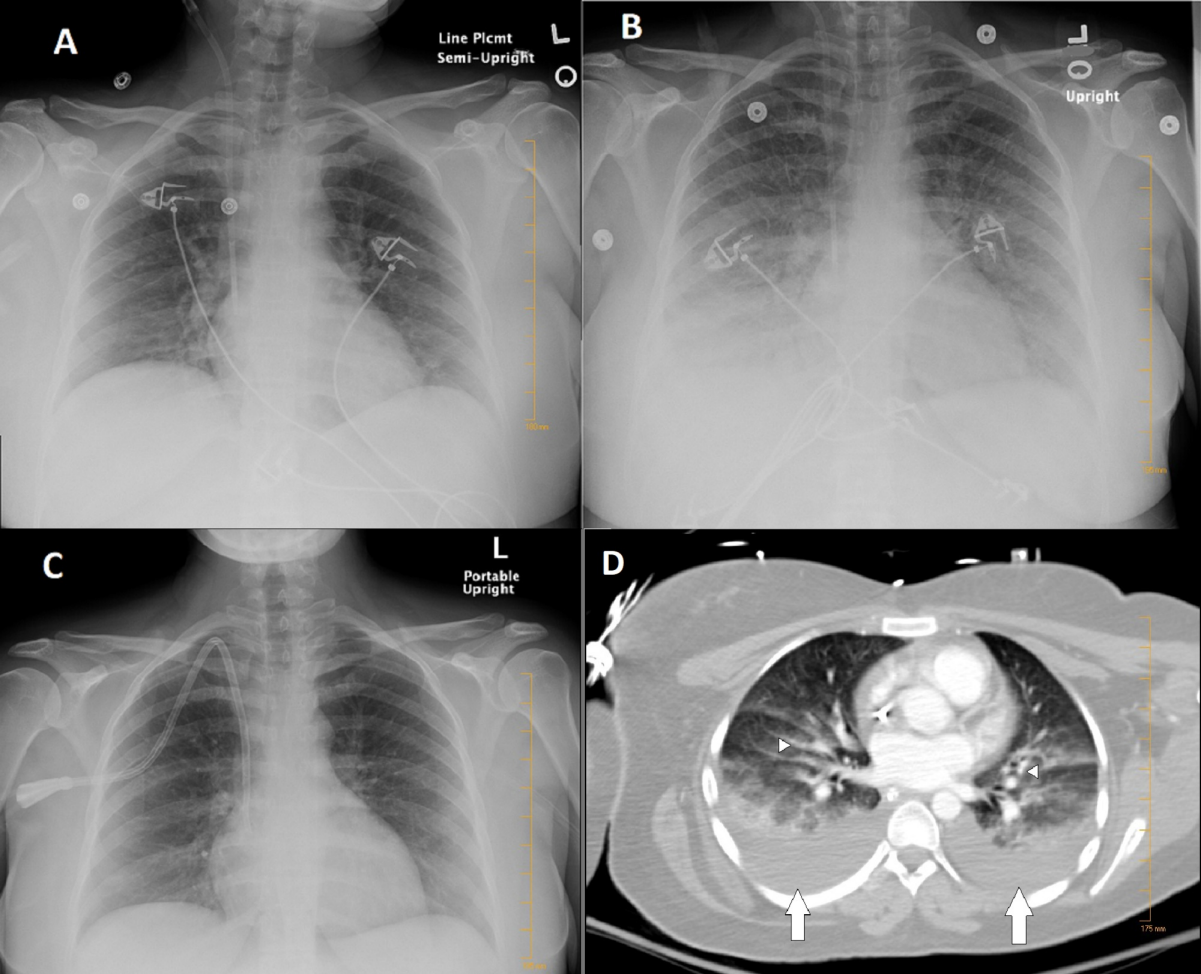
Chest radiographs and computed tomography (CT) of the patient during her hospital course A) No acute intrapulmonary process, normal cardiac size; B) bilateral perihilar interstitial opacities and vascular congestion suggestive of interstitial pulmonary edema; C) complete resolution of vascular congestion. The heart is normal in size and contour; pulmonary vessels are normal in size and caliber; D) contrast-enhanced computed tomography (CT) of the chest showing bilateral pleural effusion (white arrows) and diffuse bilateral vascular congestion (arrowheads) suggestive of pulmonary edema; no pulmonary embolism noted.

**Figure 2:**
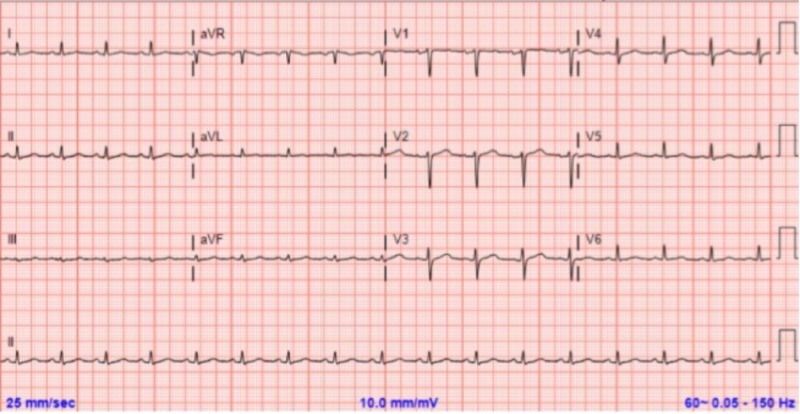
Electrocardiogram at admission without significant abnormalities QTc = 439 ms; no T wave changes

The patient's chest x-ray (CXR) was initially unremarkable (Figure 1A), as well as her ECG (Figure 2). On the following day, the patient developed progressively worsening dyspnea and hypoxemia without chest pain. The physical exam revealed tachycardia at 138 beats per minute, elevated blood pressure at 170/100 mmHg, and diffuse crackles over bilateral lungs. No murmurs were heard and no jugular venous distention was noted. CXR showed diffuse bilateral vascular congestion typical for diffuse pulmonary edema (Figure 1B); the troponin T level was elevated at 0.43 ng/mL (normal range: 0.00 - 0.01 ng/mL) but subsequently downtrended. The patient was noted to have minimal urine output over the previous few hours. Subsequently, the patient was intubated and hemodialysis initiated due to fluid overload.

**Figure 3:**
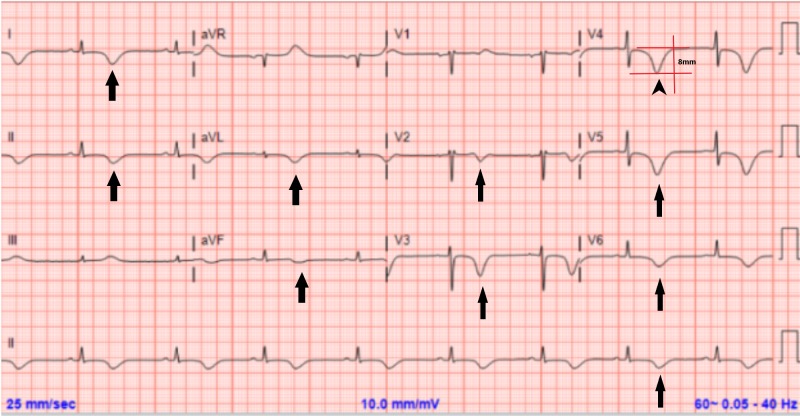
Electrocardiogram a few hours after onset of pulmonary edema symptoms QTc = 562 ms; diffuse T wave inversion (arrows) with a peak T wave amplitude of 8 mm in V4 (arrowhead)

**Figure 4:**
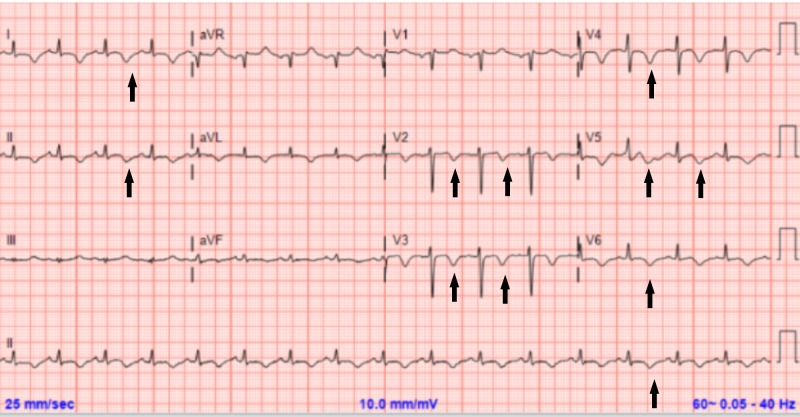
Electrocardiogram 12 hours after the resolution of symptoms QTc is 520 ms; significant decrease in the amplitude of T waves

**Figure 5:**
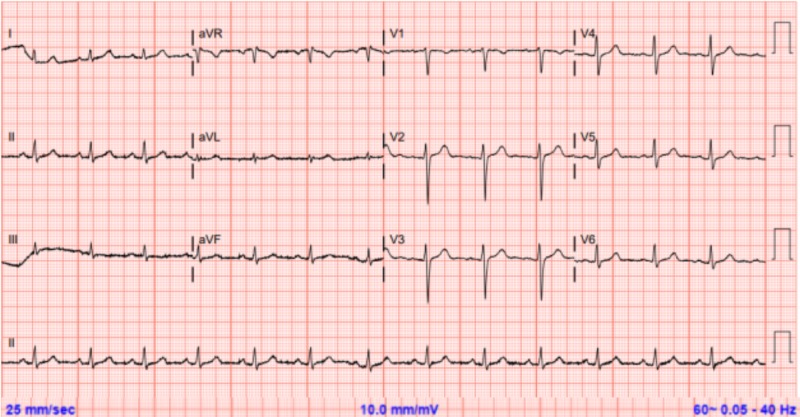
Electrocardiogram three months after discharge showing complete resolution of T wave inversion

An ECG was repeated and revealed new precordial deep T wave inversions with a prolonged QT interval (Figure 3). The patient was immediately intubated and urgent hemodialysis was performed. A contrasted computed tomography (CT) of the chest was negative for pulmonary embolism and was suggestive of pulmonary edema (Figure 1D). A transthoracic echocardiogram (TTE) revealed a normal ejection fraction of 60%, without wall motion or valvular abnormalities (Video 1). Her electrolytes were within normal limits during the period of respiratory distress. The patient was extubated the next day after the improvement of her respiratory status and resolution of pulmonary edema on follow-up CXR (Figure 1C). A repeat TTE was obtained which did not show any abnormality and was virtually the same as the first one. ECG the following day showed improved T wave inversions and shortening of the QT interval (Figure 4). She continued to improve clinically and was discharged home. Three months later, the patient was evaluated in the clinic with complete resolution of symptoms and normalization of T-wave inversions (Figure 5). In addition to that, a cardiac magnetic resonance imaging (MRI) study was done and ruled out any current or previous ischemic insult. 

**Video 1:** Two-dimensional (2D) transthoracic echocardiogram Normal 2D echocardiogram showing multiple views of the heart for a young female patient who presented with diffuse deep T-wave inversion during an episode of non-cardiogenic pulmonary edema. Left ventricle: left ventricular size was normal. Systolic function was normal. Ejection fraction was estimated to be 60%. Overall regional wall motion was normal. Left ventricular wall thickness was normal. There was no evidence of a mass or thrombus. Left ventricular diastolic function parameters were normal. Right ventricle: The right ventricular size was normal. Systolic function was normal. Wall thickness was normal. There was no evidence of a mass or thrombus. The right ventricular systolic pressure was 43 mmHg. No vegetations were noted.

## Discussion

T wave inversion has been historically classified into different categories based on the degree of inversion (mild T wave inversion (-0.1 mV to - 0.5 mV), deep negative T wave (-0.5 mV to -1.0 mV), and giant negative T wave (greater than -1.0 mV), etiology (cardiac - ischemic and non-ischemic- or noncardiac), and mechanism (primary - altering the myocardial electrophysiology by injury - and secondary - altering the conduction and ventricular activation pathways by bundle branch block or ventricular hypertrophy) [1, 3].

Giant precordial negative T waves on ECG have been linked to many cardiac and non-cardiac causes. It is now believed that this finding is less likely to be due to myocardial ischemia and is not related to the severity of coronary artery disease [4]. It is more related to anatomical changes in the heart or acute neurological catastrophes, particularly when associated with QTc prolongation. The differential diagnosis of deep and giant negative T waves is quite extensive and includes cardiac causes, such as Wellen’s syndrome (defined as precordial deep negative T waves in the setting of unstable angina and critical stenosis of the proximal left anterior descending (LAD) coronary artery [5]), new and old myocardial ischemia [6], asymmetrical apical hypertrophic cardiomyopathy [7], pericarditis, myocarditis, cardiac metastasis [8], post-pacing or ventricular conduction disturbance changes (cardiac memory) [9], congenital long QT syndromes [10], and Takotsubo cardiomyopathy [11], with less common causes, such as heart block, bradycardia, right ventricular hypertrophy, right bundle branch block, metabolic disturbances, and changes during diagnostic coronary angiography [1, 6]. Neurological causes include ischemic stroke and transient ischemic attacks (TIA) [12], subarachnoid hemorrhage [13], and electroconvulsive therapy [14]. Pulmonary causes are also reported due to pulmonary embolism [15] and pulmonary edema [16-17]. In addition, these T wave changes are also reported as a normal variant in specific leads and population [3]. 

We performed a systematic review of the literature for similar cases through Pubmed and Cochrane from 1920 to August 10, 2017, using the English language. The medical subject headings, Emtree, and keyword search terms used in combination were: T wave inversion, prolonged QT interval, negative T wave, and pulmonary edema. There was no restriction for the study type and all PubMed indexed studies were included. Two reports were found by Littmann [17] and Pascal et al [16].

A total of 12 cases of pulmonary edema associated with T wave inversion and QT prolongation were found. The case reports are summarized in Table 2.

**Table 2:** Summary of characteristics of our patient and similar cases from the literature Patients 1-9 are Littmann’s patients [17], 10-12 are patients from the Pascal et al. study [16], and patient 13 is our patient. AAH: anteroapical hypokinesia; AR: aortic regurgitation; BP adm: blood pressure on admission (mm Hg); E/A: transmitral E wave-A wave reversal; EF: left ventricular ejection fraction; F: female; LAD: left atrial dilation; LVH: left ventricular hypertrophy; M: male; MR: mitral regurgitation (4+ = severe, 2+ = moderate); QTc adm: rate-corrected QT interval in the admission electrocardiogram (ms); QTc max = the longest rate-corrected QT interval in subsequent electrocardiograms (ms); T ampl: maximum amplitude of inverted T waves in mm (10 mm/mV). * Echocardiographic findings for our patient (Video 1) Permission was obtained from Dr. Littmann to reproduce this table from his original paper in 1999 [17].

Case	Age (years)	Gender	Etiology	How CAD was excluded	ECHO; EF	BP adm	QTc adm	QTc max	T ampl
1	44	F	Rheumatic heart disease s/p mitral valve replacement.	Coronary angiography	4+MR; 0.55	170/100	449	572	11
2	79	F	Atrial fibrillation	Coronary angiography	0.30	144/60	369	544	24
3	34	F	Eclampsia	Age, absence of cardiac risk factors or chest pain. Echocardiogram.	E/A; 0.55	200/120	403	650	3
4	74	F	Hypertension, atrial fibrillation	Absence of chest pain, echocardiogram. Adenosine myocardial perfusion SPECT scan.	LVH; 0.50	164/103	441	456	6
5	32	M	Hypertension, dilated cardiomyopathy	Age, absence of cardiac risk factors or chest pain. Adenosine myocardial perfusion SPECT scan.	LVH; 0.35	203/132	480	592	6
6	72	M	Hypertension, chronic renal insufficiency.	Absence of chest pain. Adenosine myocardial perfusion SPECT scan.	LVH; 0.45	210/130	451	519	5
7	66	F	Hypertension, volume overload.	Absence of cardiac risk factors or chest pain. Dobutamine stress echocardiogram.	0.55	115/62	469	553	15
8	74	F	Hypertension, aortic regurgitation.	Absence of cardiac risk factors or chest pain. Dobutamine stress echocardiogram.	AR; LVH; 0.40	180/100	397	543	14
9	73	F	Mitral regurgitation	Coronary angiography	2+ MR; 0.60	158/103	422	604	10
10	50	F	Hypertensive crisis, volume overload	Echocardiogram showed anteroapical hypokinesis. Coronary angiography was negative.	LVH; AAH; 0.50	230/120	443	581	14
11	72	F	Hypertensive crisis	Coronary angiography	LAD, MR, AR; 0.50	220/110	435	626	20
12	60	F	Hypertensive crisis, volume overload	Absence of cardiac enzymes, echocardiogram findings.	MR; 0.45	240/160	424	458	11
13 Our case	28	F	Acute kidney injury, volume overload.	Absence of cardiac risk factors or chest pain, echocardiogram findings*.	Normal; 0.65	170/100	439	562	8

We analyzed these 12 patients. The average age was 61 years (range: 32 - 79 years). Ten of them were females and all of them had underlying cardiogenic non-ischemic etiology to the development of pulmonary edema, including hypertensive crisis, dilated cardiomyopathies, and valvular heart disease. None of these patients presented with typical chest pain, neurological emergency, or symptoms suggesting pulmonary embolism or pheochromocytoma. All other causes of T wave inversion were ruled out, including electrolyte abnormalities and ischemia. T wave inversion amplitude ranged between 3 and 24 mm and maximum QT interval was between 456 and 650 ms with females having both deeper inversions and longer maximum QT intervals. The first nine cases had diffuse and global T wave inversions, whereas the remaining three cases were reported as isolated T-wave inversions in the precordial leads. ECG changes started to develop between a few hours to days after the resolution of symptoms. In patients who underwent serial ECG follow-ups after the resolution of symptoms, a complete resolution of the ECG changes was observed. Each of the patients reported had a prolonged hospital stay due to the subsequent diagnostic workup that was done as a response to the ECG changes.

Our case is unique due to the absence of any cardiac factor as the cause of her pulmonary edema. She is a young, previously healthy female who did not suffer from any vascular, valvular, cardiac, or chronic medical illness. A cardiac MRI was also negative for ischemic changes. Contrary to the previously reported cases, there was no time lag between the onset of pulmonary edema and ECG changes and her symptoms started resolving a few days later. The significant female predominance in cases of diffuse deep and giant negative T wave continued to manifest in our patient and the reported series, as well as in the multiple previous series of deep and giant negative T wave cases [18-19]

Suggested mechanisms

The mechanism of giant and deep negative T wave on ECGs in the reported patients are not well understood. To our knowledge, there is no animal model to simulate. To understand the pathophysiology behind the findings, we suggest the following mechanisms:

Severe Stress and Excessive Catecholamines Effect

T waves on the electrocardiogram reflect the repolarization of the ventricular muscles. It is usually positive in most precordial leads due to the fact that the subepicardium repolarizes before the subendocardium [3]. Subendocardial ischemia or stress will lead to shorter action potential duration and earlier repolarization prior to the subepicardial area, hence causing the negative T wave. This is the pathophysiology behind T wave inversion during ischemia and ECG changes in patients with subarachnoid hemorrhage and stress cardiomyopathy [11, 13] which are very similar to the changes we are reporting during pulmonary edema. It is suggested that the excessive sympathetic activation during neurological catastrophes and severe stress lead to transient vasoconstriction in the intramural coronary arteries or by the direct toxic effect of catecholamines on the myocardial vasculature leading to subendocardial ischemia [20]. This mechanism can explain the T wave inversion in the reported pulmonary edema patients.

Right Ventricular Strain

The acute right ventricular strain in patients with pulmonary embolism causes severe pulmonary vasoconstriction and severe increase in pulmonary arterial pressure due to hypoxia and is the likely explanation for the ECG changes in patients with pulmonary edema with resultant severe hypoxia [15].

Myocardial Edema

Similar ECG changes are also reported in patients with myocarditis. Myocardial edema was evident on cardiac MRI in these patients [8]. Possible myocardial edema and irritation in acute pulmonary edema is a potential explanation for these changes in our patients.

## Conclusions

Even though negative T waves are considered nonspecific ECG changes and can be associated with different etiologies (as well as being found in normal healthy individuals), giant and deep negative T waves can rarely be associated with non-cardiogenic pulmonary edema. Recognizing this rare Wellen’s-like electrocardiographic pattern is crucial and plays a vital role in guiding further investigations, management, and follow-up plans, particularly in patients in whom ischemia has been ruled out. As of this time, the knowledge about these ECG changes is limited. Our article's aim is not to suggest a change of the current approach to such findings but to raise an awareness about the importance of further basic science and clinicopathological correlation, as well as prospective studies, to help understand the pathophysiology and the long-term clinical impact behind this ECG finding and to formulate electrophysiological criteria to further identify these changes earlier in the course of disease.
